# Intervene before leaving: clustered lot quality assurance sampling to monitor vaccination coverage at health district level before the end of a yellow fever and measles vaccination campaign in Sierra Leone in 2009

**DOI:** 10.1186/1471-2458-12-415

**Published:** 2012-06-07

**Authors:** Lorenzo Pezzoli, Ishata Conteh, Wogba Kamara, Marta Gacic-Dobo, Olivier Ronveaux, William A Perea, Rosamund F Lewis

**Affiliations:** 1 Consultant for the World Health Organization, Geneva, Switzerland; 2 Immunization and Vaccine Development, World Health Organization, Freetown, Sierra Leone; 3 Statistics Sierra Leone, Freetown, Sierra Leone; 4 Immunization Vaccines and Biologicals, World Health Organization, Geneva, Switzerland; 5 Immunization and Vaccine Development, World Health Organization, Ouagadougou, Burkina Faso; 6 Epidemic Readiness and Intervention, World Health Organization, Geneva, Switzerland

**Keywords:** Clustered lot quality assurance sampling (C-LQAS), Measles vaccine, Yellow fever vaccine, Vaccination coverage, Monitoring, Africa, Sierra Leone

## Abstract

**Background:**

In November 2009, Sierra Leone conducted a preventive yellow fever (YF) vaccination campaign targeting individuals aged nine months and older in six health districts. The campaign was integrated with a measles follow-up campaign throughout the country targeting children aged 9–59 months. For both campaigns, the operational objective was to reach 95% of the target population. During the campaign, we used clustered lot quality assurance sampling (C-LQAS) to identify areas of low coverage to recommend timely mop-up actions.

**Methods:**

We divided the country in 20 non-overlapping lots. Twelve lots were targeted by both vaccinations, while eight only by measles. In each lot, five clusters of ten eligible individuals were selected for each vaccine. The upper threshold (UT) was set at 90% and the lower threshold (LT) at 75%. A lot was rejected for low vaccination coverage if more than 7 unvaccinated individuals (not presenting vaccination card) were found. After the campaign, we plotted the C-LQAS results against the post-campaign coverage estimations to assess if early interventions were successful enough to increase coverage in the lots that were at the level of rejection before the end of the campaign.

**Results:**

During the last two days of campaign, based on card-confirmed vaccination status, five lots out of 20 (25.0%) failed for having low measles vaccination coverage and three lots out of 12 (25.0%) for low YF coverage. In one district, estimated post-campaign vaccination coverage for both vaccines was still not significantly above the minimum acceptable level (LT = 75%) even after vaccination mop-up activities.

**Conclusion:**

C-LQAS during the vaccination campaign was informative to identify areas requiring mop-up activities to reach the coverage target prior to leaving the region. The only district where mop-up activities seemed to be unsuccessful might have had logistical difficulties that should be further investigated and resolved.

## Background

**Figure 1:**
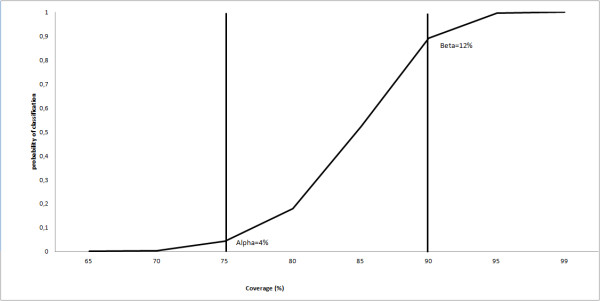
**Operating characteristic curve for LQAS rule rejecting programmes with more than 7 defectives in a sample of 50 assuming simple random sampling (no clustering).** Two vertical lines are placed at the lower and upper thresholds.

Lot Quality Assurance Sampling (LQAS) is a technique used by the World Health Organization (WHO) Expanded Programme on Immunization (EPI) to evaluate vaccination programmes [1]. The LQAS test is based on two thresholds: the lower threshold (LT), which is the minimum vaccination coverage level considered to be acceptable, and the upper threshold (UT), which is generally the operational objective of vaccination activities (i.e. the vaccination coverage target) [2]. Based on these thresholds, a decision value (d), i.e. the maximum number of unvaccinated individuals allowed in the sample (N) to classify the lot as with acceptable vaccination coverage, is calculated. If the number of unvaccinated individuals detected is equal or below *d,* the lot is classified as having an acceptable coverage (i.e. “accepted”), meaning that coverage in the lot is probably equal or above the LT. If the number of unvaccinated is above *d,* the lot is classified as having an unacceptable coverage (i.e. “rejected”), meaning that coverage in the lot is probably below the UT. Alpha, calculated from the LT, is the probability of “accepting” a lot with low coverage; while beta, calculated from the UT, is the probability of “rejecting” a lot with high coverage. When the real coverage in the lot falls in the “grey area” delimited by UT and LT, alpha and beta increase, making the LQAS classification less precise [3-5]. The LQAS rule can be summarized by the operative characteristic (OC) curve (Figure 1).

LQAS requires smaller sample sizes and offers the advantage that the survey can be stopped if *d* is exceeded in the lot. It is therefore quicker than the standard vaccination coverage cluster-sampling survey[6]; however, it does not allow estimation of vaccination coverage with 95% confidence interval at the lot level. The "classic" LQAS approach is based on simple random sampling (SRS) rather than cluster-sampling [7]. Using SRS on large lots, such as health districts, introduces logistic challenges and engenders costs as the sample is scattered over a wide geographic area [8]. Clustered Lot Quality Assurance Sampling (C-LQAS) is a modified design of LQAS, which allows for the lots to be divided into smaller clusters, considerably increasing rapidity of implementation in the field [2,9]. C-LQAS employs the same statistical principles as the standard LQAS, with the difference that *N* is divided in clusters (k) of *n* individuals. Statistical precision is then recalculated assuming that the true coverage in each cluster would vary from the mean lot coverage according to a binomial distribution with preset standard deviations (SD) [2]. Several authors have explored the applications of dividing the sample in smaller clusters, while applying sequential sampling techniques, such as LQAS, to different fields, from the assessment of global acute malnutrition [10-12] to applications in clinical audit [13], veterinary medicine [14], or agriculture [15,16]. WHO has been piloting C-LQAS with Ministries of Health (MoH) in west and central Africa to monitor coverage while vaccination campaigns are in progress in order to identify areas that need mop-up activities before leaving the field [17].

From 24 to 29 November 2009, Sierra Leone conducted a preventive yellow fever (YF) vaccination campaign among individuals aged nine months or older in six of its 14 health districts: Western Area Urban, Western Area Rural, Koinadugu, Kambia, Tonkolili and Port Loko. The campaign was integrated with a measles follow-up campaign throughout the country, among children aged 9–59 months. For both campaigns, the operational objective was to reach 95% of the target population.

We used C-LQAS to identify areas in Sierra Leone with unacceptably low yellow fever and/or measles vaccination coverage during the last two days of the ongoing vaccination campaign in order to improve coverage in the areas of weakness before the end of vaccination activities.

## Methods

### Study population

The study population was the target population for each vaccination campaign, as estimated through annual projections of district populations from the 2004 census in Sierra Leone [18]. For YF vaccination, the target population was 2 435 690 for individuals aged nine months and older (excluding pregnant women) living in the six participating districts. For measles vaccination, the target population was 824 366 children aged 9–59 months living in all 14 districts of Sierra Leone.

We defined an individual vaccinated against YF as a person aged nine months or more, presenting the YF vaccination card from the current campaign or documented proof of prior vaccination (such as previous YF campaigns). We defined an individual vaccinated against measles as a child aged 9–59 months, presenting the measles vaccination card from the current campaign.

We also recorded if the individuals or their caregivers verbally reported that they had received the measles or YF vaccines during the campaign, but we did not use this information to take operational decisions in the field.

### Definition of lots

**Figure 2:**
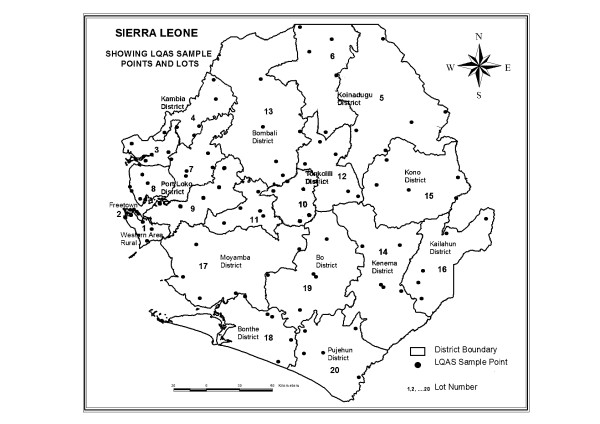
Clustered lot quality assurance sampling vaccination coverage survey, with twenty lots and five random sampling points per lot, Sierra Leone, 28–29 November 2009.

Sierra Leone is divided into four provinces: the Western Area (WA) and the Northern, Southern and Eastern Provinces. WA is divided into two districts (Urban and Rural), each of which is divided into wards and then further divided into enumeration areas (EAs) comprised of different localities (i.e. villages or neighbourhoods). The remaining provinces are divided into districts, then into chiefdoms, and finally into EAs [18]. We divided the fourteen districts of Sierra Leone into 20 non-overlapping lots (Figure 2). We considered each of the eight health districts targeted only for measles vaccination as a lot. We divided the six health districts targeted for both measles and YF into 12 smaller lots, based on the population size of the chiefdoms and their geographic location, so to have sub-district lots of approximately the same population size (Table 1).

**Table 1:** List of lots per district for the LQAS survey, Sierra Leone, 28–29 November 2009

**District**	**Vaccination**	**Lot Number**	**Chiefdoms**	**Population**	%*
Western Area Rural	YF + M	1	Entire District	169807	100
Western Area Urban	YF + M	2	Entire District	764484	100
Kambia	YF + M	3	Magbema + Mambolo + Samu	157807	58.4
		4	Bramaia + Gbinle Dixing + Masungbala + Tonko Limba	112569	41.6
Koinadugu	YF + M	5	Mongo + Neya + Nieni + Sengbe + Sulima	144347	54.3
		6	Dembelia Sinkulia + Diang + Folosamba Dembelia + Kasunko + Wara Wara Bafodia + Wara Wara Yagala	121336	45.7
Port Loko	YF + M	7	BKM + Dibia + Maforki + Sanda Magbolontor + TMS	151198	33.4
		8	Kaffu Bullom + Lokomasama	141374	31.2
		9	Buya Romende + Koya + Marampa + Masimera	160447	35.4
Tonkolili	YF + M	10	Gbonkolenken + Kholifa Rowala + Tane	117298	33.9
		11	Yoni + Kholifa Mabang + Malal Mara	113828	32.8
		12	Kafe Simiria + Kalansongoia + Kunike + Kunike Barina + Sambaya	115330	33.3
Bombali	M	13	Entire District	394603	100
Kenema	M	14	Entire District	463410	100
Kono	M	15	Entire District	293660	100
Kailahun	M	16	Entire District	348781	100
Moyamba	M	17	Entire District	255466	100
Bonthe	M	18	Entire District	139605	100
Bo	M	19	Entire District	448961	100
Pujehun	M	20	Entire District	225373	100

YF: Yellow Fever; M: Measles.

* Proportion of district population in the lot.

### Lot sample size

We calculated possible C-LQAS plans for use in the field by setting the UT at 90 or 95% and the LT at 75 or 80%, respectively. For feasibility reasons we used N = 50, divided into 5 clusters of 10. Using computer simulations previously described [2], we modelled how alpha and beta would change if the coverage between the clusters was set to vary up to 0.1 SD from the mean coverage in the lot (Table 2).

**Table 2:** Clustered lot quality assurance sampling plan used to evaluate yellow fever and measles vaccination coverage, Sierra Leone, 28–29 November 2009

***N***	***d***	**%LT**	**%UT**	**Clusters*k*x*n***	**SD range**	**% Alpha range**	**% Beta range**
50	4	80	95	5x10	0-0.1	2-5	10-19
50	7	75	90	5x10	0-0.1	4-8	12-19
100	15	75	90	10x10	0-0.1	1-3	4-12
150	25	75	90	15x10	0-0.1	1-4	1-4

N: lot sample size; d: decision value; LT: lower threshold; UT: upper threshold; k: number of clusters; n: cluster sample size; SD: standard deviation.

We decided to use the sampling plan with UT = 90%, basing this decision on previous experiences indicating that coverage during the last two days of campaign would be at 90% and not yet at 95% [Central command data team of the MoH, personal communication]. Similarly, LT was taken as 75% and *d* was calculated as seven (Table 2).

### Sampling

In each lot we selected five EAs using probability proportional to population size (PPS) [19]. In each EA, we randomly selected one locality by simple random ballot to start the survey. Once in the selected locality, we divided it into quarters of approximately the same size according to existing divisions (streets, rivers, etc.), using a map (sketched on site if unavailable). We selected one quarter by simple random ballot using the table of random numbers. If there were fewer than 20 houses in the quarter, we numbered them and selected one randomly as the starting point of the survey. If the houses were more than 20, we divided the quarter into further smaller quarters, until we were able to randomly select one with less than 20 houses. If the compound or building selected contained more than one household (e.g. a building occupied by several families), we numbered them and selected one household to survey by simple random ballot. Finally, we numbered all the eligible individuals per household and selected one by random ballot to respond to the survey. If the individuals randomly selected were eligible for both vaccines, we administered both questionnaires; if they were eligible only for YF, after administering the YF questionnaire, we proceeded to a second random selection only in the 9–59 months age group to administer the measles questionnaire. If the selected individual was under ten years of age, we asked a parent or an older caregiver to answer the questions on the child’s behalf. We did not conduct the interview and moved to next household, if there was nobody in the house above 10 years old.

We always moved to the right of the household to select the subsequent ones, leaving a gap of four households for the YF survey and two for the measles survey in rural areas. In the urban areas, we left a gap of eight houses for YF and four for measles. If we reached the end of the locality without having surveyed ten individuals, we moved to the geographically closest locality in the same lot and repeated the procedure to complete the cluster.

Since the campaign was in progress, we obtained information from district medical officers about the localities already reached by campaign activities. At the same time, we did not disclose the exact location of the five clusters. We surveyed the localities not previously reached by vaccination activities only on the second day of survey (i.e. last day of the campaign), when every locality should have been covered.

### Data collection and corrective measures

We used standardized questionnaires for data collection. For the purpose of taking action based on documented evidence before the end of the campaign, we considered any individual not presenting a vaccination card as not vaccinated. As soon as *d* was reached in the lot, we recommended the survey teams to inform the district medical officers, so that immediate mop-up actions could be taken while vaccination teams were still in the field. We also recommended a one-day extension of the vaccination campaign in the rejected lots. Instead of stopping once *d* was reached, we completed the sample in every lot, since we wanted to conduct further analysis on the complete sample. We also aimed to avoid the potential source of bias due to the fact that surveyors might have more actively looked for unvaccinated individuals to finish the survey faster.

### Inter-cluster variability

In each lot, we calculated the standard error (SE) of vaccination coverage between the five clusters assuming a two-stage clustered design sampled with PPS. We then used the calculated SE as a measure of inter-cluster variability (ICV) to verify if it exceeded the maximum SD of 0.1 from the mean lot coverage assumed in the C-LQAS plan.

### Design effect

We calculated the maximum design effect (DEFF), assuming coverage at UT or LT. We estimated what the variance would be, if the sample would have been based on simple random sampling, by deriving the standard error (SE) from the assumed coverage from the sample size (n = 50) according to the following formula, described elsewhere [20]:

(1) $$\text{SE}=\surd \left[\text{p}\left(1-\text{p}\right)/\text{n}\right]$$

We then derived the maximum assumed DEFF as the ratio of the maximum assumed variance of the clustered sample (in our case, SD = 0.1) to the variance of a simple random sample of the same number of elements [21] for the vaccination coverage at 75% or 90%:

(2) $$\text{DEFF}={\left(\text{variance in clustered design}\right)}^{2}/{\left(\text{variance in SRS design}\right)}^{2}$$

### End-campaign and post-campaign vaccination coverage

Results from a series of 30x14 cluster-sampling surveys conducted two months after the campaign (from the 9^th^ to the 20^th^ of January 2010) to estimate vaccination coverage were available for nine districts [Campaign Evaluation Team, MoH/WHO/UNICEF, Unpublished Data]. To assess if control measures were successful to increase coverage levels in the lots that were rejected for low coverage before the end of the campaign, we plotted the C-LQAS results against the 95% confidence intervals of the post-campaign coverage estimations, considering verbal history of vaccination. We used the confidence intervals of the coverage estimates as thresholds for statistical significance, assessing if post-campaign coverage was significantly above 75%. In the districts that had been divided in more than one lot, in order to obtain a C-LQAS classification at district level that could be checked against the post-campaign coverage estimate, we combined the lot samples and calculated C-LQAS plans with UT = 90% and LT = 75% (Table 2).

## Results

### Measles vaccination coverage

**Figure 3:**
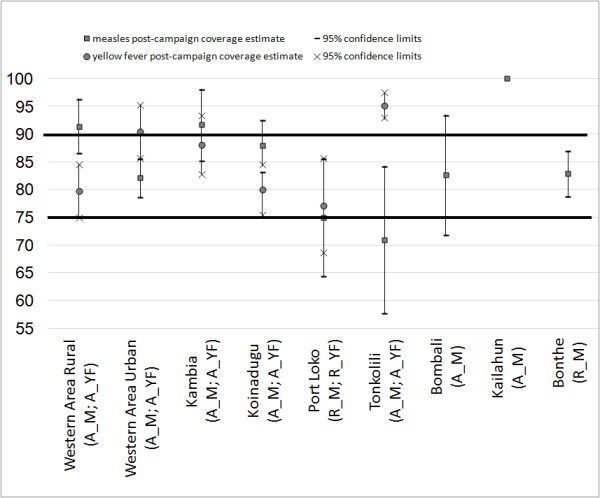
Results of the end-campaign clustered lot quality assurance sampling decision with the results of the post-campaign vaccination coverage* with 95% confidence interval, obtained at district level by considering verbal history of vaccination, Sierra Leone, November 2009 - January 2010. Legend: A_M: Accepted by the LQAS rule for measles vaccination coverage; R_M: Rejected by the LQAS rule for measles vaccination coverage; A_YF: Accepted by the LQAS rule for yellow fever vaccination coverage; R_YF: Rejected by the LQAS rule for yellow fever vaccination coverage. *Post campaign coverage estimations provided by the Campaign Evaluation Team, MoH/WHO/UNICEF (Personal Communication).

During the last two days of campaign, 75.0% (15/20) of lots were accepted as having adequate (≥75%) measles coverage based on card-confirmed vaccination status; by considering also verbal history of vaccination 80.0% (16/20) were accepted (Table 3). Five of the seven districts accepted for measles vaccination coverage before the end of the campaign, presented an estimated post-campaign coverage significantly above 75%. Among the two rejected districts, Bonthe presented post-campaign measles coverage significantly above 75%, while in Port Loko post-campaign coverage was not significantly above 75% [Campaign Evaluation Team, MoH/WHO/UNICEF, Unpublished Data] (Figure 3).

**Table 3:** Results of the clustered lot quality assurance sampling surveys^*^to monitor measles vaccination coverage before the end of the campaign, Sierra Leone, November 2009

**District**	**Lot**	**N**	**Vaccination Card-Verified**^§^			**Verbal History**		
			**U**	**D**	**SE**	**U**	**D**	**SE**
Western Area Rural	1	50	4	A	0.04	0	A	0.00
Western Area Urban	2	50	7	A	0.07	0	A	0.00
Kambia	3	50	1	A	0.02	0	A	0.00
	4	50	2	A	0.02	0	A	0.00
	District Total	100	3	A	0.02	0	A	0.00
Koinadugu	5	50	11	R	0.07	1	A	0.02
	6	50	16	R	0.18	8	R	0.07
	District Total	100	27	R	0.09	9	A	0.03
Port Loko	7	50	18	R	0.21	15	R	0.16
	8	50	5	A	0.06	5	A	0.06
	9	50	11	R	0.15	10	R	0.15
	District Total	150	34	R	0.09	30	R	0.08
Tonkolili	10	50	1	A	0.02	0	A	0.00
	11	50	2	A	0.04	2	A	0.04
	12	50	1	A	0.02	0	A	0.00
	District Total	150	3	A	0.02	2	A	0.01
Bombali	13	50	6	A	0.06	1	A	0.02
Kenema	14	50	1	A	0.02	0	A	0.00
Kono	15	50	0	A	0.00	0	A	0.00
Kailahun	16	50	3	A	0.04	3	A	0.04
Moyamba	17	50	5	A	0.04	5	A	0.04
Bonthe	18	50	8	R	0.14	8	R	0.14
Bo	19	50	5	A	0.05	0	A	0.00
Pujehun	20	50	2	A	0.02	2	A	0.02

N: sample size; U: number of Unvaccinated in the sample; D: Decision; A: Accepted; R: Rejected; SE: Standard Error of the distribution of the vaccination variable between the clusters in the lots.

* Sampling plans used: UT = 90% and LT = 75%, with d = 7 when N = 50 (5x10); d = 15 when N = 100 (10x10); d = 25 when N = 150 (15x10).

§ Used to take operational decisions in the field before the end of the campaign.

### Yellow fever vaccination coverage

During the last two days of campaign, 75.0% (9/12) of lots were accepted as having adequate YF coverage (≥75%) based on card-confirmed vaccination status; by considering also verbal history of vaccination 83.3% (10/12) of lots were accepted (Table 4). All five districts accepted by the end-campaign C-LQAS rule, presented post-campaign YF vaccination coverage significantly above 75%. Port Loko, rejected because of a lower than expected YF end-campaign coverage, did not present post-campaign coverage significantly above 75% [Campaign Evaluation Team, MoH/WHO/UNICEF, Unpublished Data] (Figure 3).

**Table 4:** Results of the clustered lot quality assurance sampling surveys^*^to monitor yellow fever vaccination coverage before the end of the campaign, Sierra Leone, 28–29 November 2009

**District**	**Lot**	**N**	**Vaccination Card**^§^			**Verbal History**		
			**U**	**D**	**SE**	**U**	**D**	**SE**
Western Area Rural	1	50	2	A	0.02	1	A	0.02
Western Area Urban	2	50	4	A	0.05	4	A	0.05
Kambia	3	50	0	A	0.00	0	A	0.00
	4	50	0	A	0.00	0	A	0.00
	District Total	100	0	A	0-00	0	A	0.00
Koinadugu	5	50	6	A	0.06	4	A	0.05
	6	50	10	R	0.07	8	R	0.08
	District Total	100	16	R	0.05	12	A	0.05
Port Loko	7	50	18	R	0.19	14	R	0.17
	8	50	7	A	0.12	7	A	0.12
	9	50	11	R	0.09	7	A	0.04
	District Total	150	36	R	0.08	28	R	0.07
Tonkolili	10	50	2	A	0.02	1	A	0.02
	11	50	2	A	0.02	2	A	0.02
	12	50	1	A	0.02	0	A	0.00
	District Total	100	5	A	0.01	3	A	0.01

N: sample size; U: number of Unvaccinated in the sample; D: Decision; A: Accepted; R: Rejected; SE: Standard Error of the distribution of the vaccination variable between the clusters in the lots.

* Sampling plans used: UT = 90% and LT = 75%, with d = 7 when N = 50 (5x10); d = 15 when N = 100 (10x10); d = 25 when N = 150 (15x10).

§ Used to take operational decisions in the field before the end of the campaign.

### Inter-cluster variability

The SE of the distribution of the variable “presenting the measles vaccination card” between the clusters in the lots exceeded 0.1 in four lots (20.0%), all of which failed according to the end-campaign LQAS rule (Table 2). The SE of the distribution of the variable “presenting the YF vaccination card” between the clusters in the lots exceeded 0.1 in two lots (16.6%), of which one passed and one failed according to the end-campaign LQAS rule (Table 3).

### Design effect

If the variation of coverage between clusters is assumed to be 0.1 SD from the mean lot coverage, DEFF would be 2.68 at 75% coverage, and 5.66 at 90%.

## Discussion

Using C-LQAS to assess vaccination coverage near the end of the integrated measles and YF campaign in Sierra Leone, we identified five areas (lots) with unacceptably low measles vaccination coverage and three with low YF coverage, based on documented proof of vaccination (card). This allowed the health authorities to put in place control measures to increase vaccination coverage before the end of the campaign in the rejected areas. Relying on verbal confirmation of vaccination status, fewer lots would have failed, as it is expected in these settings [17,22]. With one exception, verbally confirmed district vaccination coverage estimated through the post-campaign cluster-sampling surveys was above the minimum acceptable threshold (75%) used for the end-campaign C-LQAS survey, suggesting that control measures to raise coverage may have been effective in the areas of weakness identified. The fact that one district (Port Loko), failing at the end of the campaign for both low measles and YF coverage, presented post-campaign measles and YF coverage figures not significantly above 75%, suggests that it may have structural problems in reaching the target population with vaccination activities.

We used C-LQAS for end-campaign monitoring. While this approach allows time to implement mop-up activities, undertaking an assessment before the end of the time allocated to achieve the target (95%) may lead to underestimation of coverage [17]. For this reason we set the UT of the C-LQAS plan to 90% instead of 95%, the actual coverage target of the campaign. We then decided to use 75% as LT based on previous experiences showing that using a “grey area” of 15% is a possible solution to keep statistical errors reasonably low if N = 50 (5x10) [17]. By setting alpha (≤8%) lower than beta (≤19%), we gave priority to the classification based on the LT (75%), rather than the UT (90%). We wanted to be more confident that we were not accepting lots with low coverage, which is a risk for the population (alpha is also known as “consumer risk”); rather than failing lots with high coverage, which is a risk for the vaccination programme (beta is also known as “provider risk”). Results of the C-LQAS surveys need to be interpreted as an indication that, towards the end of the campaign, at least 92% (1-alpha) of the accepted lots were probably reaching the minimum acceptable coverage level of 75%; while at least 81% (1-beta) of the failed lots were probably not reaching the desirable coverage level of 90%. In other words, when we accept a lot we are 92% confident that real coverage is at least 75% (i.e. when we reject we cannot be confident that coverage is 75%).

Intense efforts from WHO and UNICEF (United Nations Children’s Fund) to encourage 90% coverage for first dose measles vaccine by 12 months of age and offering a second opportunity for vaccination have contributed to a substantial reduction of measles cases in the African region [23,24]. In this respect, the results (i.e. 15 lots out of 20 accepted for measles coverage at least at 75%), albeit before the end of the campaign, are far from optimal. Although achieving 75% vaccination coverage may be enough to stop transmission of YF, it is definitely not enough for measles, for which the threshold believed to confer herd immunity is 95% [25]. If we would have used the other, stricter, decision value proposed in Table 2 (d = 4; UT = 95%; LT = 80%) we would have accepted even fewer lots (i.e. 10 out of 20) as with measles coverage at least at 80%. By allowing individuals to present only the vaccination card of the current campaign as evidence of measles vaccination we may have underestimated the actual measles coverage in the country. Even if there was an underestimation of the true coverage rates, it is important to note that three weeks before the vaccination campaign the largest measles outbreak over the last decade started in the country and lasted until July 2010, confirming that measles vaccination coverage was low in Sierra Leone [26].

The C-LQAS approach works better if there is indication that the lots are homogeneous in terms of coverage [2,9,17]. Where possible, we attempted to divide districts into smaller lots to increase the likelihood of homogeneity. The distribution of vaccination coverage between the clusters in the lots exceeded the maximum assumed 0.1 SD always in rejected lots, apart from one occasion, Lot 8 in Port Loko, which had adequate YF coverage. Such a high ICV of vaccination coverage indicates that some of the clusters in those lots had many unvaccinated individuals, while others had few (i.e. the lot is not homogeneous in terms of vaccination coverage). This kind of distribution of coverage may indicate that some of the EAs may have been missed or inappropriately covered by vaccination activities (a good reason to reject the lot for poor quality of vaccination).

We showed that assuming that vaccination coverage would vary according to a SD of 0.1 in the clusters is a similar approach to assuming a DEFF of approximately three (if coverage is at 75%) or approximately six (if coverage is at 90%). We believe that this is more conservative than assuming DEFF = 2 as it is done in a standard manner while designing cluster surveys when there is no previous information to guide this decision [6]. The fact that the assumed DEFF increases proportionally to the assumed coverage is explained by the fact that assuming a variance of 0.1 SD when coverage is high (i.e. 90%) has a greater magnitude than assuming the same variance when coverage is low (i.e. 75%). Therefore, our approach should be more robust as the levels of real coverage get higher.

This study is subject to a number of limitations. First, because of limited resources we conducted the C-LQAS surveys at sub-district level only in the areas of the country targeted by both vaccines. In the part of the country targeted only for measles vaccination, each district was a lot. These districts have a mean total population of approximately 350,000. It may have been difficult to implement targeted control measures in such large districts. To overcome this limitation we advised the surveying teams to discuss the results of the C-LQAS surveys during the daily campaign meetings chaired by the district medical officers, in order to facilitate the implementation of control measures using all the information available also from other sources (e.g. vaccination teams or campaign supervisors). Second, by using PPS to select clusters, we inevitably placed data collection in the most populous communities. These are areas that generally are easier to cover also by vaccinators, so vaccination coverage may have been overestimated. PPS does not yield a spatially even sample as can be seen in Figure 2. Alternative methods based on geographic sampling could be used to allow that sampling locations are more evenly spread across the lots [27-29]. Third, as previously seen with the C-LQAS approach, the high inter-cluster variability (SE > 0.1) seen in some lots, reaching a maximum level of SE = 0.21, may indicate that the plans may have had errors (alpha and beta) above the levels defined in some lots [9,17], although this finding should be interpreted with caution, since it is not possible to support it statistically given the small sample sizes per lot used to calculate the SE. One way to reduce the inter-cluster variability and consequently increase the precision would be to increase the number of clusters sampled to more than five [22]. Finally, when we aggregated several lots to obtain a district level LQAS classification, we were not able to exactly assess how the unequal probabilities of selection (i.e. different lot population sizes) may have affected alpha and beta. On the other hand, the increased sample sizes at district level combining more than one lot would probably have errors that are lower than the ones assumed at lot level.

## Conclusions

Identifying lots with low vaccination coverage before the end of the campaign allowed the health authorities to put in place timely control measures to increase vaccination coverage while vaccination activities were still ongoing. Post-campaign coverage estimations indicated that control measures to raise coverage were likely to have been effective in the areas of weakness identified, although areas of low coverage still remained in the country. Dividing the districts into smaller lots increased the particularity of the assessment, enabling us to locate areas with unvaccinated populations in smaller geographic or administrative units. The decision to use 75% as LT should be revised when assessing measles vaccination coverage. Further studies could compare C-LQAS results with those of cluster-sampling surveys conducted in the same areas at the same time, to validate statistically the use of C-LQAS in alternative.

## Competing interests

The survey was co-funded by the WHO Yellow Fever Initiative, under a grant from the GAVI Alliance and the WHO Immunization, Vaccines and Biologicals Department. The authors declare that they have no competing interests.

## Authors’ contributions

LP, OR and RFL had the idea for the study. LP, IC, and WK have written the study protocol. MGD, OR, WP, and RFL revised the study protocol. LP, IC, and WK conducted the study in the field with assistance from MGD. LP and MGD collated the data. The analyses were done by LP, who also drafted the paper. All authors commented on the draft and approved the final version.

## Pre-publication history

The pre-publication history for this paper can be accessed here:

http://www.biomedcentral.com/1471-2458/12/415/prepub
